# Ultrastructure Analysis and Molecular Characterization of *Trichomitus batrachorum* (Parabasalia; Hypotrichomonadida) Isolated from Liver of *Ameiva ameiva* (Reptilia: Squamata)

**DOI:** 10.3390/microorganisms13061286

**Published:** 2025-05-31

**Authors:** Lina Maria Pelaez Cortes, Júlia de Castro Ascenção, Rhagner Bonono dos Reis, Gabriela Peixoto, Gabriel Gazzoni Araújo Gonçalves, Jana Messias Sandes, Fábio André Brayner dos Santos, Luiz Carlos Alves, Felipe Arley Costa Pessoa, Claudia María Ríos Velásquez, Helena Lúcia Carneiro Santos

**Affiliations:** 1 Oswaldo Cruz Foundation, Laboratory of Ecology of Transmissible Diseases in the Amazon, Post-Graduation Program in Biology of Hosped Pathogen Interação, Leônidas and Maria Deane Institute, Manaus 69057-070, AM, Brazil; lcortes@aluno.fiocruz.br (L.M.P.C.); gabriela.peixoto@fiocruz.br (G.P.); felipe.pessoa@fiocruz.br (F.A.C.P.); 2 Oswaldo Cruz Foundation, Laboratory of Parasitic Diseases, Oswaldo Cruz Institute, Rio de Janeiro 21040-900, RJ, Brazil; julia.ascencao@ioc.fiocruz.br (J.d.C.A.); rhagner.bonono@ioc.fiocruz.br (R.B.d.R.); 3 Electron Microscopy Laboratory, Keizo Asami Institute (iLIKA), Federal University of Pernambuco, Recife 50670-901, PE, Brazil; gabriel.gazzoni@ufpe.br (G.G.A.G.); jana.sandes@ufpe.br (J.M.S.); fabio.brayner@fiocruz.br (F.A.B.d.S.); luizcarlos.alves@fiocruz.br (L.C.A.); 4 Cellular and Molecular Biology Laboratory, Department of Parasitology, Aggeu Magalhães Institute, FIOCRUZ-PE, Recife 50.740-465, PE, Brazil

**Keywords:** biodiversity, Amazon rainforest, trichomonadids, climate change, *Ameiva*

## Abstract

*Trichomitus batrachorum* is a species of trichomonad that has gained attention due to its ecological importance and potential interactions with various hosts, such as amphibians (anurans) and reptiles (lizards and chelonians), where it has been recorded in the gastrointestinal tract of these vertebrates, specifically in their feces. Molecular studies have placed this flagellated protist within the Metamonada clade. Unlike parabasalids that inhabit endothermic mammals in relatively stable temperature conditions, protists associated with ectothermic reptiles are subject to significant temperature fluctuations. The ability of *T. batrachorum* to thrive in the variable temperatures encountered by reptiles suggests that its parasitism may remain largely unaffected by climate change. In our study, we detected and characterized *T. batrachorum* from the liver tissue of the lizard species *Ameiva ameiva*, collected in Presidente Figueiredo Municipality, Amazonas State, Brazil. The identification of *T. batrachorum* was confirmed by cultivation technique, light microscopy, scanning electron microscopy and transmission electron microscopy for ultrastructural analyses, and sequencing the 5.8S rDNA (region ITS1- ITS2) and 18S rRNA (ribosomal RNA) genes. One potential interpretation for this finding is that the flagellates may have migrated from the intestine to the bile duct, ultimately reaching the liver. This is the first successful characterization of *T. batrachorum* in the liver of a lizard, and provides a solid foundation for further research to elucidate the potential pathogenicity of this flagellate and the role of *A. ameiva* in the epidemiology of parabasalids in other animal species.

## 1. Introduction

*Trichomitus batrachorum* is a species of trichomonad protist classified within the Excavata supergroup, specifically in the Metamonada clade and the phylum Parabasalia [1]. This species has garnered interest due to its significant ecological roles and potential interactions with various hosts, including amphibians and reptiles.

Molecular analyses have positioned this flagellated protist within the order Hypotrichomonadida and the family Hypotrichomonadidae. Phylogenetic analyses based on ribosomal RNA sequences have shown that *T. batrachorum* is closely related to *Hypotrichomonas acosta*, suggesting a complex evolutionary history within the trichomonads [1,2,3]. This relationship elucidates its evolutionary connections with other trichomonads. The genii *Trichomitus* and *Hypotrichomonas* belong to the smallest parabasalian class, Hypotrichomonadea, which is characterized by the presence of three anterior flagella, a lamelliform undulating membrane, a biramous parabasal body, and a cytoskeletal structure known as a comb-like structure in the mastigont [4].

*Trichomitus batrachorum* is identified by its elongated, pear-shaped (pyriform) flagellated form, which typically features three to five anterior flagella and a single recurrent flagellum. This recurrent flagellum functions as an undulating membrane, facilitating movement through aquatic environments and enhancing the organism’s ability to capture prey [5].

The size of *T. batrachorum* generally ranges from 10 to 30 μm in length, classifying it among the smallest trichomonads [6]. Its shape can vary slightly depending on environmental conditions and the physiological state of the organism. Under unfavorable conditions, it can survive in the form of pseudocysts. This dormant state involves the internalization of flagella and other organelles, allowing the organism to endure adverse environments [7].

Ultrastructural studies using transmission electron microscopy (TEM) have revealed additional details about the internal organization of *T. batrachorum*. The organism possesses a well-developed pelta (a structure associated with the flagellar apparatus), a V-shaped parabasal body, and a prominent axostyle, a rod-like structure that provides support [8]. The pelta–axostylar complex and the costa are the main cytoskeleton structures observed. Numerous glycogen granules and some large vesicles and hydrogenosomes are present in the cytoplasm. The marginal lamellae and the recurrent flagellum constitute the undulating membrane. The accessory filament is a segment of the fold distal to the recurrent flagellum [9]. Extra-axonemal structures have also been noted, suggesting a potential role in the organism’s motility and structural integrity, a fascinating area for future research [10].

Interestingly, while many trichomonads do not form true cysts, *T. batrachorum* has been observed to have a cyst wall, a significant feature among some members of the Hypotrichomonadida. This cyst wall is typically thick and may exhibit a filamentous appearance, providing additional protection to the organism [9,11].

Parabasalians are obligate flagellated organisms and commonly found in the gastrointestinal and genitourinary systems of humans and various animals. Trichomonadids in animal hosts are transmitted by the fecal–oral route [12,13]. *T. batrachorum*, primarily isolated in the gut of reptiles [9], is also described as a frog symbiont [14], and has been found in various mammals (pigs and primates), suggesting potential zoonotic risks [2,5]. It is important to mention that the pigs from which *T. batrachorum* was isolated did not show any symptoms of disease [14].

Reptiles are considered important reservoirs of several protists with medical and veterinary relevance, like *Giardia duodenalis*, *Cryptosporidium parvum*, *Sarcocystis nesbitti*, *Leishmania* sp. and *Trypanosoma brucei* [15]. These parabasalid species have been associated with the bile ducts and gallbladder in reptiles, possibly due to migration from the gastrointestinal tract, which in some cases can cause significant inflammation of the biliary system [16]. The parabasalians’ proliferation in reptiles may be due to immunosuppression caused by pathological processes, resulting in anorexia, diarrhea, polydipsia, and weight loss [12,17,18].

*Trichomitus batrachorum* has been described in different reptiles (Reptilia, Squamata) [19]. *Ameiva ameiva* is a reptile of the Teiidae family, which is well known due to its wide distribution and adaptability to modified environments throughout its geographical distribution in Central, Caribbean and South America, where it is found at altitudes from 23 m to 2000 m, in open areas, such as grasslands, savanna, rural and urban habitats, and in tropical rain and dry forests, Yungas forests, and seasonally flooded forests [20,21].

The objective of this study was to record for the first time the unusual occurrence of *T. batrachorum* in the liver of *A. ameiva*, and add characteristics through isolation, light microscopy, SEM, TEM, and sequencing techniques. This reported case highlights the importance of biodiversity studies that allow us to determine the microfauna of vertebrates, and the potential risks to human and animal health, especially considering with the actual landscape and climate change.

## 2. Materials and Methods

### 2.1. Ethical Statement

All procedures were approved by the Chico Mendes Institute for Biodiversity Conservation (ICMBio/SISBIO) (license #87315-1) and by the Ethics Committee on Animal Use (CEUA) of the CEUA Fiocruz Rondônia (CEUA RO #2019/21). All experiments were conducted in accordance with CONCEA (National Council for Animal Experimentation Control) guidelines. The project was registered in the National System for the Management of Genetic Heritage and Associated Traditional Knowledge—Sisgen (AFE0154).

### 2.2. Samples and Location

**Figure 1:**
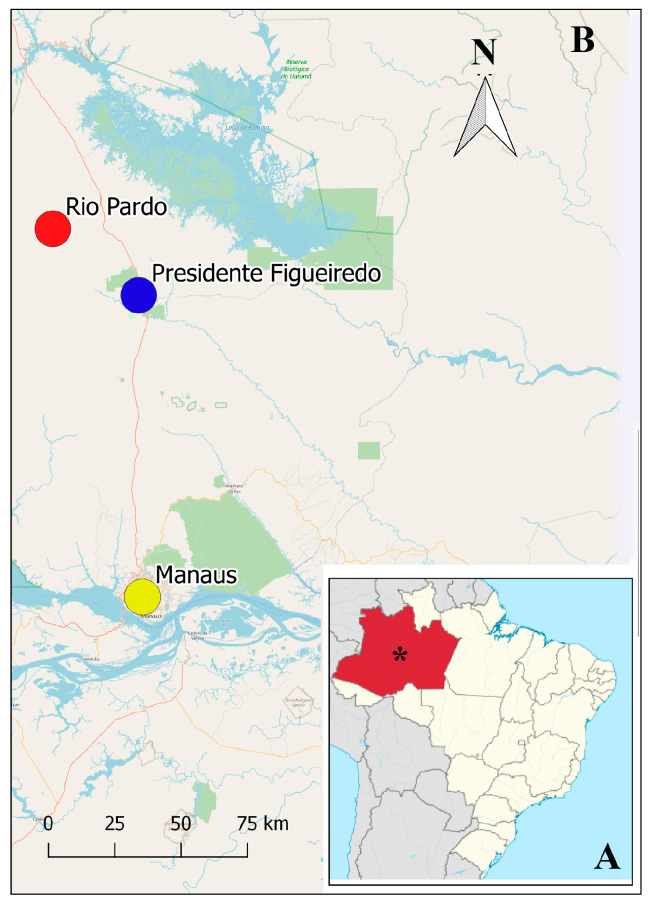
Map of Brazil showing (**A**) the Amazonas State (in red) and Manaus city with *, and in the increased area (**B**), Manaus Municipality (yellow circle), the municipality of Presidente Figueiredo (blue circle) and Rio Pardo Rural Settlement (red circle).

Reptiles were collected between 2023 and 2024 in the Rio Pardo Rural Settlement, located in Presidente Figueiredo, AM (S 01°49′02.4″ W 60°19′03.6″), a municipality 110 km away from Manaus (Figure 1). The criteria for selecting the animals that were part of the study were that they were adult reptiles; hatchlings and juvenile animals were excluded. The reptiles were identified using the keys for lizard identification [22]. The reptile infected with *T. batrachorum* was collected on 8 March 2024, during an unusual dry period.

The captured reptiles were anesthetized by the intraperitoneal injection of ketamine hydrochloride (100 mg/kg) and xylazine hydrochloride (10 mg/kg), according to procedures recommended by the National Council for Animal Experimentation Control (CONCEA).

After the induction of anesthesia, blood was collected by cardiac puncture and preserved in sterile polypropylene tubes with three distinct media. Subsequently, all specimens were sacrificed by increasing the anesthetic dose to at least three times the usual dose [23]. Liver fragments were collected by laparotomy and placed into separate sterile polypropylene tubes with three distinct media. Subsequently, the individuals were tagged, fixed in 10% formalin, and preserved in 70% methanol [24].

All animals were stored at the Zoological Collections of the National Institute of Amazonian Research (INPA) in Manaus, Brazil.

### 2.3. Isolation and Cultivation

The initial and most pivotal step in our study involved the isolation and culturing of blood and liver tissue samples. These samples were cultivated in three distinct media: (a) Novy–McNeal–Nicolle in conjunction with Liver Infusion Tryptose (NNN/LIT), (b) NNN/Schneider, and (c) NNN/SNB-9. All media were supplemented with 10% fetal bovine serum (FBS) and 5% antibiotics (penicillin and streptomycin). This medium was kept refrigerated until use (stable for 2 to 4 weeks) and left at room temperature before inoculation and during field activities. The final pH of the medium was 7.0 ± 0.2 (at 25 °C). After use, it was maintained in BOD at a temperature of 27–28 °C for approximately 15 days. The positive sample was successfully isolated from a piece of liver cultivated in the supplemented LIT medium, representing a significant achievement in our research. The parasite was fixed with methanol before being submitted to Giemsa staining.

### 2.4. Scanning Electron Microscopy (SEM)

*Trichomitus batrachorum* cultivations were washed in a buffer solution (0.1 M sodium cacodylate, pH 7.2), and placed in Eppendorf tubes for later fixation in 0.1 M sodium cacodylate buffer, 2.5% glutaraldehyde and 4% formaldehyde. Then, post-fixation was performed in 1% osmium tetroxide in 0.1 M cacodylate buffer for 90 min, protected from light. Afterwards, three washes were performed in 0.1 M cacodylate buffer for the subsequent dehydration of the worm specimens, using an increasing sequence of ethanol at 30%, 50%, 70% and 90%, and 3 times at 100%, for 10 min each wash. After the process of dehydration, the critical point phase was performed for the replacement of ethanol with carbon dioxide. The equipment used was the Critical Point Dryer, CPD 030, BAL-TEC (Balzers, Austria), Technological Platforms Nucleus of the Aggeu Magalhães Institute—FIOCRUZ/PE. After this step, the dry material was removed from the equipment and sent to be mounted on metal stubs using double-sided carbon tape. Finally, the samples were metallized in an atmosphere with a thin layer of gold for visualization and analysis in the scanning electron microscope (MEV—5600 LV—JOEL) from the Technological Platforms Nucleus of the Aggeu Magalhães Institute—FIOCRUZ/PE.

### 2.5. Transmission Electron Microscopy (TEM)

*Trichomitus batrachorum* cultivations were washed in 0.1 M phosphate buffer (pH 7.4), centrifuged for 524 g and fixed in Karnovski (0.1 M phosphate buffer and 2.5% glutaraldehyde) (Sigma Aldrich, St. Louis, MO, USA) for 60 min, followed by three washes in 0.1 M phosphate buffer for 10 min, and then post-fixation in 1% osmium tetroxide (Electron Microscopy Science, Hatfield, PA, USA) and 0.1 M phosphate buffer (pH 7.2) for 90 min. Dehydration was performed with acetone (Sigma Aldrich) and infiltration with Epon 812 resin (Electron Microscopy Science) to perform ultrathin cuts, which were contrasted with uranyl acetate and lead citrate (Electron Microscopy Science) and observed under a transmission electron microscope (FEI Tecnai^TM^ Spirit G2 BioTWIN) from the Technological Platforms Nucleus of the Aggeu Magalhães Institute—FIOCRUZ/PE.

### 2.6. Molecular Characterization

DNA was extracted from the cultures using the Wizard^®^ SV Genomic DNA Purification System Kit (Promega Corporation, Madison, WI, USA), according to the manufacturer’s instructions. The extracted DNA was stored at −20 °C until amplification via polymerase chain reaction (PCR).

We used the primers for trichomonads ITSF (5’-TTCAGTTCAGCGGGTCTTCC-3’), ITSR (5’-GTAGGTGAACCTGCCGTTGG-3’) for 5.8S rDNA (region ITS1—ITS2) [5,25], and 18SF (5’-AATCTGGTTGATCCTGCCAG-3’) and 18SR (5’-TGATCCTTCTGCAGGTTCACCTA-3’) primers for the 18S rRNA (ribosomal RNA) genes [2].

The PCR reaction, totaling 50 µL, consisted of 3 µL of DNA solution, 25 µL of Promega PCR Master Mix (Promega Corporation, Madison, WI, USA), and 1.25 µL of each primer solution. The PCR cycling conditions included 1 cycle at 94 °C for 5 min, followed by 30 cycles at 94 °C for 1 min, 57 °C for 1 min, and 72 °C for 1.5 min, with a final extension at 72 °C for 7 min (1 cycle). PCR products were visualized on a 1.5% agarose gel containing Nancy-520 from Sigma-Aldrich^®^ under UV light. The PCR products were purified using the Wizard^®^ SV Gel and PCR Clean-up System, following the manufacturer’s instructions.

### 2.7. Sequencing and Phylogenetic Analysis

DNA cycle sequencing reactions were performed using the BigDye^®^ Terminator v.3.1 Cycle Sequencing Kit and loaded in the ABI 3730 Sequencing Platform (both Applied Biosystems, Foster City, CA, USA). Sequence alignment and consensus were performed using the SeqMan program from the DNASTAR software package https://www.dnastar.com/ (DNASTAR Inc., Madison, WI, USA). These sequences were then uploaded to the NCBI BLAST (V. 2.2.15) tool (https://blast.ncbi.nlm.nih.gov/Blast.cgi (accessed on 16 October 2024)) to identify similar sequences within known datasets. Additionally, other published sequences of various trichomonads were downloaded from GenBank, and multiple sequence alignments were conducted using the Multiple Alignment using Fast Fourier Transform (MAFFT—https://mafft.cbrc.jp/alignment/server/index.html (accessed on 17 October 2024)). To determine the phylogenetic position of the isolates, phylogenetic trees were constructed using *Trichomonas vaginalis* as the outgroup. Two probabilistic methods were employed for this purpose—Maximum Likelihood (ML) and Bayesian Inference (BI). Both methods utilized the GTR + F model (General Time Reversible model with unequal rates and unequal base frequencies) and were implemented using the PhyloSuite v1.2.3 software algorithm [26,27].

The nucleotide sequences reported in this paper were deposited in GenBank under PQ771696.1.

## 3. Results

**Figure 2:**
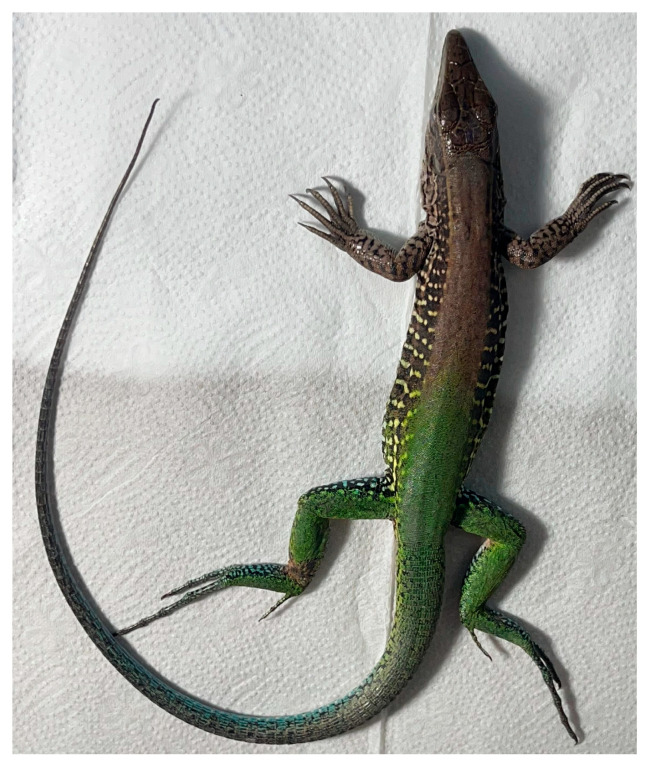
*Ameiva ameiva* Reptilia: Squamata.

The two most frequently encountered species were *A. ameiva* (Figure 2), comprising 36.5% of the total (15 out of 41), and *Tupinambis teguixin*, which accounted for 24% (10 out of 41).

The main result of this study was the isolation and cultivation of *T. batrachorum* from liver tissue fragments of the reptile *A. ameiva* in cultivation medium. The reptile was a unfertilized adult female.

**Figure 3:**
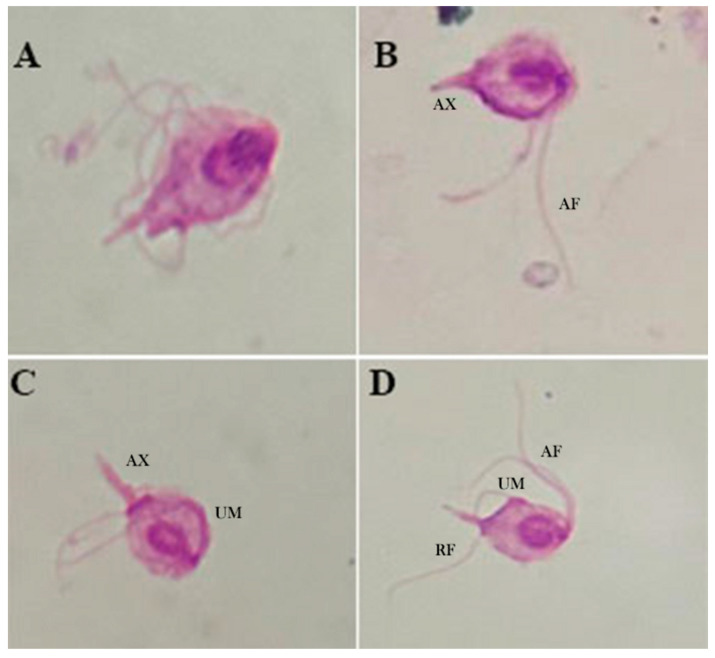
Light microscopy (100×) of Giemsa-stained *T. batrachorum* isolated from liver tissue fragment of *A. ameiva* in cultivation medium. (**A**,**B**) Piriform form of the cell, the anterior flagella (AF) with unequal length, and the axostyle (AX). (**C**) The axostyle (AX) is clearly observed, with a pointed structure and the undulating membrane (UM). (**D**) Anterior flagella (AF) and the recurrent flagellum (RF) situated next to the undulating membrane (UM).

Light microscopy. Under light microscopy, trichomonads from the axenic culture showed an ovoid shape with a pointed posterior tip that coalesces at its axostyle, undulating membranes, and the presence of an undetermined number of flagella. Irregular, spasmodic, and rapid locomotion, often with a wave-like motion, could be observed, which is consistent with findings from previous studies, and helps confirm the observations made in our study (Figure 3).

**Figure 4:**
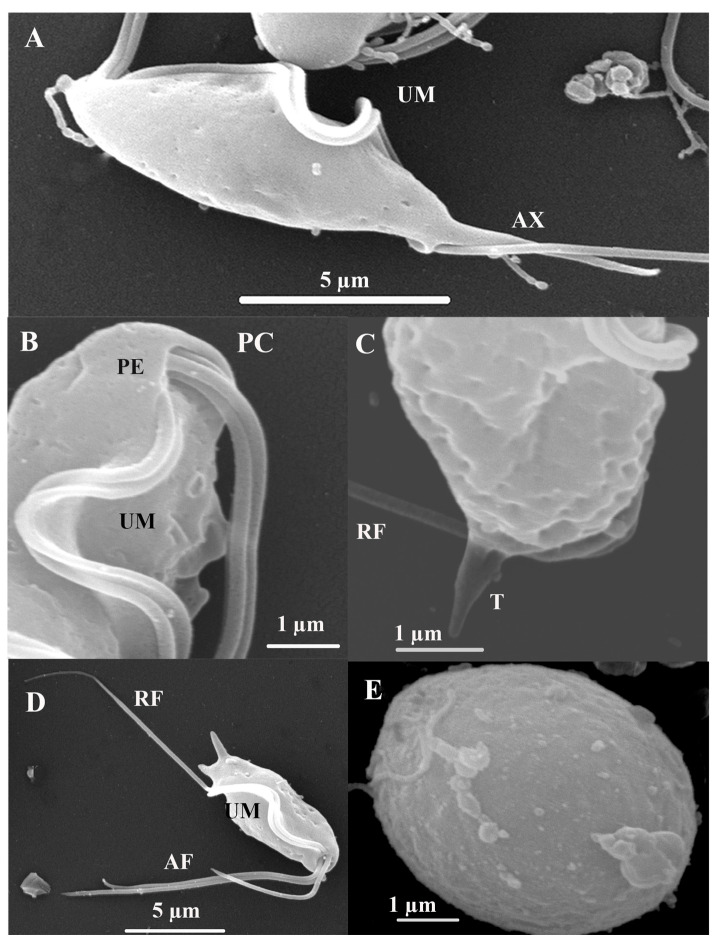
*Trichomitus batrachorum* SEM: (**A**–**D**) Anterior flagella (AF), recurrent flagellum (RF) undulating membrane (UM), the axostyle (AX), pelta (PE), t tail-like terminal part (T) and periflagellar canal (PC). (**E**) cyst. F (TEM) Photomicrograph of *Trichomitus batrachorum*.

SEM. The surface structure of T. *batrachorum* reveals a pyriform cell. The free external margin of the undulating membrane (UM) includes the attached segment of the recurrent flagellum (RF), which extends freely for approximately one-third of its total length, and the three anterior flagella (AF) of unequal lengths originating from the periflagellar canal (PC). Additionally, the structure includes the axostyle (AX), which has a hyaline structure and expands to a point, and the pelta (PE); these are part of the main cytoskeleton structures observed. We also observed that the three anterior flagella have unequal lengths, and that these originate from the periflagellar canal (PC). It is important to note a cyst stage form, which is circular and doer not present flagellar structures (Figure 4).

**Figure 5:**
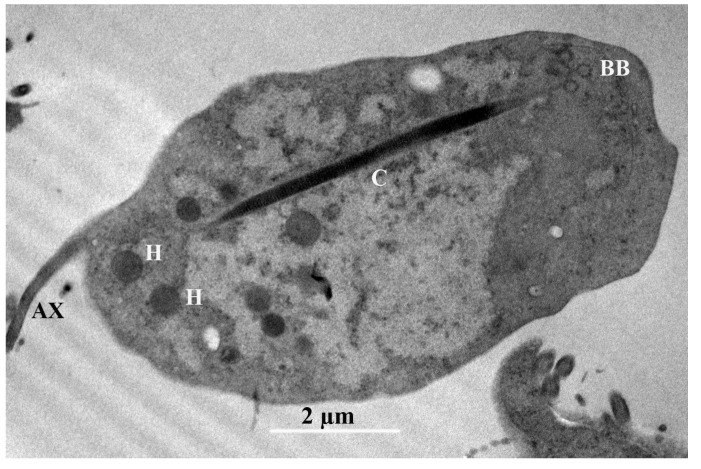
*Trichomitus batrachorum* TEM: axostyle (AX), hydrogenosomes (H), the costa (C) and the parabasal body (BB).

TEM. In the transmission microphotograph, we can observe a section of the costa (C), the axostyle (AX), and numerous glycogen granules, along with some large vesicles and hydrogenosomes (H), in the cytoplasm. The glycogen granules are distributed throughout the cytoplasm of the cell and have a dark color. The Golgi complex or parabasal body (BB) is a microfibrillar strand, and the parabasal body consists of peripherally vesiculated cisternae. We observe here a costa with a cytoskeletal structure found only in trichomonads, and assume that this provides mechanical support to the undulating membrane. It is a large, striated root fibril that begins in the basal body (BB) region of the recurrent flagellum, extending toward the posterior region of the cell (Figure 5) [28].

The 18S rRNA gene sequence, consisting of 1342 nucleotide positions, and the ITS sequence, which includes 345 base pairs, were successfully obtained for analysis. The ITS sequence was used to construct the corresponding phylogenetic tree, which groups the trichomonad protozoan sequences into well-supported branches according to their genera. The dendrogram based on ITS sequence data includes *Hypotrichomonas acosta*, which belongs to the same family as *T. batrachorum*, followed by the free-living species *Ditrichomonas honigbergii* and the genus *Monocercomas*, which was isolated from a snake. The following groups belong to the genus *Tritrichomonas*, and occured in different mammalian hosts. These were followed by the genera *Tetratrichomonas* spp., *Pentatrichomonas*, and *Trichomonas*, which originated from domestic animals, and *Trichomonas gallinaae*, which originated from poultry.

**Figure 6:**
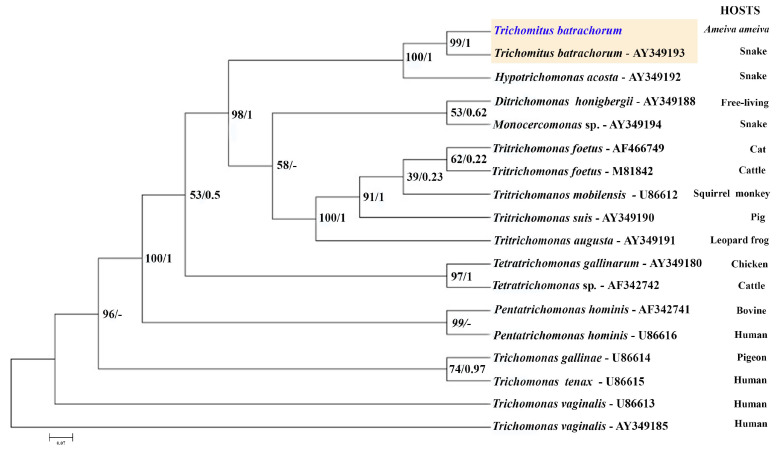
Maximum likelihood (ML) and Bayesian inference reconstruction tree of *T. batrachorum* based on the 5.8S rDNA (ITS1–ITS2 region) gene (345 bp), using a sequence obtained in the present study (highlighted in blue) and retrieved from Genbank. The first number associated with each node represents the ML bootstrap value (values below 50% are not shown) followed by the Bayesian posterior probabilities. All the strains, except AF466749, AF342742 and AF342741, were obtained from the American Type Culture Collection (ATCC), Rockville, MD, USA (AF466749: animal shelter, North Carolina USA; AF342742 and AF342741: University of California, Davis, CA, USA).

The ITS-1/5.8S/ITS-2 region of ribosomal DNA has indeed proven to be a valuable and reliable locus for phylogenetic studies within the Trichomonadida, particularly at lower taxonomic levels such as species and genera, and even up to the family level (Trichomonadidae) [29]. While the ITS region is excellent for its lower-level phylogenetic resolution, it is worth noting that for deeper relationships within the Trichomonadida (e.g., at higher taxonomic levels like orders or subphyla within Parabasalia), other more conserved genes like the small subunit ribosomal RNA (SSU rRNA or 18S rRNA) or protein-coding genes are often employed to avoid the saturation of the ITS region with too many mutations (Figure 6). The DNA sequences of the isolate showed remarkably high identity rates of 99.35% for the 18S rRNA gene region (JX565060.1) and 99.15% for the ITS sequence (AY349193.1) for *T. batrachorum* isolated from snakes. The samples were clustered with *T. batrachorum* GenBank sequences, demonstrating strong bootstrap support and Bayesian posterior probabilities.

## 4. Discussion

Our research has led to the isolation of *T. batrachorum* from liver samples cultured in two different media. This is a significant achievement, as it is the first record of this flagellate being isolated from a reptile’s liver, and the first record of its presence in the Brazilian Amazon. The description of this finding was carried out meticulously, with a key role played by SEM and TEM, and it coincides with previous studies, which mentioned the form and the same structures in the external structure [9]. It is important to note a cyst stage that was observed in this study, because the trichomonads typically only have trophozoite and pseudocyst forms, but *T. batrachorum* also exhibits this form [9].

Regarding morphological features, three species were reported that belong to the *Trichomitus batrachorum* complex (*Ts. batrachorum*) due to morphological similarities and unknown physiological differences: *Trichomonas batrachorum*, *Tritrichomonas batrachorum* (Perty) and *Trichomonas natricis* Coutelen, Biguet and Cochet. These may actually be synonyms of *Trichomitus batrachorum* (Perty) [30,31]. Currently, two genera are recognized in the Hipotrichomonadidae family: *Hipotrichomonas* and *Trichomitus* [3].

When comparing with SEM findings of *T. foetus* in axenic cultures, it is possible to observe a teardrop shape, three anterior flagella and a recurrent flagellum, similar to *T. batrachorum*. In the case of the cell of *T vaginalis,* it is less pear-shaped than *T. foetus*, and it has an anterior flagellum and recurrent flagellum too. The structures observed in *T. Batrachorum* with TEM are similar to those observed in *T. vaginalis* and *T. foetus*, including hydrogenosomes, axostyle, basal bodies, the nucleus and the pelta [28].

These techniques, along with molecular methods, allow us to derive the sequences and, through phylogenetic analyses, confirm the species of the organism using the ITS1 region (ITS1-5.8S rDNA ITS2) and 18S rRNA (ribosomal RNA) genes. These markers have been instrumental in the description of Parabasalids organisms, including *T. batrachorum* [2,5,9,25,29].

*Trichomitus batrachorum* has been identified in feces, rectal swabs, and intestinal tissue samples from a variety of hosts, including amphibians, reptiles, and mammals, such as rodents, pigs, and primates [2,4,9,13]. The organism has also been found in captive reptiles in California, USA, as well as in wild reptiles in Colombia, indicating its presence as part of the reptilian gut microbiota [19]. The occurrence of *T. batrachorum* across such a diverse range of hosts prompts significant inquiries regarding its potential implications for animal health. Furthermore, parabasalids have been detected in the bile ducts and gallbladder, likely due to migration from the gastrointestinal tract [16]; however, there are currently no reports of its presence in liver fragments.

Notably, other species of parabasalids have been documented in the livers of birds. For example, *Tetratrichomonas gallinarum*, which is typically found in the intestinal tract, has been associated with diseases such as hepatitis and typhlitis, especially when more virulent strains are involved [25,32]. Similarly, *Trichomonas gallinae* has been isolated from the livers of pigeons, with evidence suggesting that it may migrate to the duodenum and subsequently travel through the bile ducts to the liver. To elucidate the pathway by which the parasite reaches the liver, an experiment was conducted involving birds that underwent esophagotomization. The findings indicate that *T. gallinae* can access the liver from the upper digestive tract via a route distinct from the alimentary canal and bile ducts [33].

In the present study, the macroscopic analysis of the liver of *A. ameiva* showed no signs of disease, abnormalities, or characteristics different from normal tissue. The route by which *T. batrochorum* reached the liver is uncertain; this issue should be investigated, as well as the possible source. In the literature, *T. batrochorum* is described as a microorganism that belongs to the microfauna of amphibians and reptiles [9]. It has the potential to infect mammals such as pigs, rodents, and non-human primates, demonstrating a wide range of hosts and the possibility of not only residing in the gastrointestinal tract, but also migrating to other organs, which can give it a zoonotic potential [5].

Regarding the proteolytic activity that has been determined in different parabasalids, *T. batrachorum* showed some similarities to *Trichomonas vaginalis* (Donné) and *Tritrichomonas foetus* (Riedmueller); however, the proteinase patterns of *T. vaginalis* and *Tt. foetus* were more similar to each other than they were to *T. batrachorum*. These findings may reflect differences in pathogenicity and habitat [34].

The pathogenic potential of parabasalids has only been observed in a few species, such as cattle and birds, neither of which live in ectothermic animals. On rare occasions, some parabasalids have been observed in moribund snakes kept in captivity and sacrificed for diagnostic purposes, as well as during midgut autopsies of spontaneously dying hosts [31].

As part of some descriptions of trichomonads, pathological changes were observed in the lungs of *a Crotalus atrox* (snake) from Mexico after a brief period of captivity. This suggests that invasions of these flagellates could occur by chance in extraintestinal organs. Liver lesions have also been reported in *Rana pipiens* (amphibia), associated with the species *Tritrichomonas augusta* (family Tritrichomonadidae), but in this case, true pathogenicity could not be demonstrated experimentally. Studies on the possible (potentially facultative) pathogenicity of certain flagellates in their ectothermic hosts are needed [31].

Although *T. batrochorum* has not been reported to cause signs of disease, the other genus of the Hypotrichomonadidae family, *Hypotrichomonas*, has been reported as causing diarrhea in a captive snake [35].

*Hypotrichomonas* spp. has been reported in non-human primates, reptiles, rodents, and deer [36]. As mentioned above, *T. batrachroum* has been reported in amphibians and reptiles, and in the following mammals, without exhibiting any symptoms: pigs, non-human primates, and rodents [2,14]. This could indicate that this class of parabasalids can inhabit a wide range of hosts, adapting to the temperatures of endothermic and ectothermic animals, but the scarce information on the pathogenic functions of Hypotrichomonads makes it difficult to determine their effects on these hosts, so more research is required to understand their life cycles and pathogenic potential [2,13].

Trichomonads have demonstrated a capability of adapting to new hosts, migrating to another site in the host, and zoonotic transmission [2,37]. Thus, it is important to continue research on these parabasalids so as to understand the possible threats to both humans and animals, and to define the pathogenic potential of these trichomonads in more detail.

It is essential to recognize that reptiles are ectothermic organisms, making them susceptible to fluctuations in temperature. Climate change has a profound negative impact on the density and diversity of reptile species, which in turn affects their microbiota [37]. Parabasalids, such as *T. batrachorum*, play a role in the reptile microbiota, and have demonstrated an ability to endure a broad range of temperatures, including cooler conditions, compared to their mammalian counterparts. However, research indicates that exposure to colder temperatures can alter the transcriptomes of protists and increase the expression of genes that may lead to detrimental interactions with their hosts [19].

In studies involving lizards subjected to elevated temperatures, it has been shown that warming can significantly disrupt and destabilize the intestinal microbiota of these reptiles. Additionally, the findings indicate a correlation between the composition of the intestinal microbiota and the host’s thermal tolerance [37]. Consequently, the ongoing climate change impacting the Amazon’s biome may have implications for the parabasalids present in the region’s herpetofauna.

The extreme temperatures currently affecting this region may negatively impact the protists, which could, in turn, affect the associated vertebrate populations. The ecological factors driving spillover and the emergence of diseases in novel hosts, including humans, involve a complex interplay of processes. Therefore, understanding the factors that facilitate the transmission of microfauna from wild animals to humans is essential for developing effective strategies aimed at reducing the frequency of spillover events.

## 5. Conclusions

This report is the first to describe *Trichomitus batrachorum* in an extraintestinal organ; specifically, the liver. The method by which the parasite accesses this organ is unknown. Understanding this mechanism could improve our understanding of the parasite’s pathogenicity. Its ability to adapt to different temperatures and access different hosts could pose a significant threat to various wild and domestic vertebrates. This report emphasizes the need for further research on these protists, and for attempts at linking it to the changes that may occur due to the pressures affecting the Amazon and fluctuations in environmental temperature.

## Data Availability

The nucleotide sequences reported in this paper were deposited in GenBank under PQ771696.1.
